# Idiopathic Inflammatory Myopathies: Clinical Approach and Management

**DOI:** 10.3389/fneur.2016.00064

**Published:** 2016-05-20

**Authors:** Asma Malik, Ghazala Hayat, Junaid S. Kalia, Miguel A. Guzman

**Affiliations:** ^1^Neurology, Saint Louis University, Saint Louis, MO, USA; ^2^Department of Neurology and Neurotherapeutics, The University of Texas Southwestern, Dallas, TX, USA; ^3^Department of Pathology, Saint Louis University, Saint Louis, MO, USA

**Keywords:** idiopathic inflammatory mypathies, polymyositis, dermatomyositis, necrotizing myopathy, inclusion body myositis, antiSRP, myositis specific antibodies

## Abstract

Idiopathic inflammatory myopathies (IIM) are a group of chronic, autoimmune conditions affecting primarily the proximal muscles. The most common types are dermatomyositis (DM), polymyositis (PM), necrotizing autoimmune myopathy (NAM), and sporadic inclusion body myositis (sIBM). Patients typically present with sub-acute to chronic onset of proximal weakness manifested by difficulty with rising from a chair, climbing stairs, lifting objects, and combing hair. They are uniquely identified by their clinical presentation consisting of muscular and extramuscular manifestations. Laboratory investigations, including increased serum creatine kinase (CK) and myositis specific antibodies (MSA) may help in differentiating clinical phenotype and to confirm the diagnosis. However, muscle biopsy remains the gold standard for diagnosis. These disorders are potentially treatable with proper diagnosis and initiation of therapy. Goals of treatment are to eliminate inflammation, restore muscle performance, reduce morbidity, and improve quality of life. This review aims to provide a basic diagnostic approach to patients with suspected IIM, summarize current therapeutic strategies, and provide an insight into future prospective therapies.

## Introduction

The Idiopathic inflammatory myopathies (IIM) are a heterogeneous group of rare systemic diseases that leads to muscle weakness, muscle enzyme elevations, inflammation on muscle biopsy, and extra muscular manifestations (1, 2).

The IIM are classified on the basis of patterns of presentation, age of onset, immunohistopathologic features, and response to treatment (3–6).The major types of IIM include: DM, PM, NAM, and sIBM. Within the last decade, NAM was made a separate subtype and was previously off-classed with PM. There is an increased risk of malignancy in specific subtypes of DM, PM, and NAM (7, 8).

DM, PM, and NAM are usually responsive to immunotherapies. sIBM is typically refractory to these agents. Since IIM are potentially treatable, proper diagnosis and early initiation of therapy are necessary (1, 2, 9–11). Consensus does not exist among experts regarding therapy and management (12).

## Classifications

Over the course of time, several different criteria have emerged to classify the IIM. Bohan and Peter criteria are the earliest and still widely used (3) (Table 1).

**Table 1:** **Diagnostic criteria for IIM**.

Criteria			Comments		
BOHAN AND PETER CRITERIA FOR DIAGNOSIS OF PM AND DM (3, 4)					
1. Symmetrical weakness of limb girdle muscles 2. Elevated levels of muscle enzymes 3. Myopathy on EMG 4. Muscle biopsy evidence of inflammation 5. Skin rash in the case of DM			• Very simple • Most widely known and used • Very sensitive • Least specific • Can only be used for DM and PM		
**DALAKAS (2003) CRITERIA FOR PM, MYOPATHIC DM AND AMYOPATHIC DM (2)**					
**Criteria**	**PM**		**Myopathic DM**		**Amyopathic DM**
	**Definite**	**Probable**	**Definite**	**Probable**	**Definite**

Myopathic muscle weakness	Yes	Yes	Yes	Yes	No
Electromyographic findings	Myopathic	Myopathic	Myopathic	Myopathic	Myopathic or non-sepcific
Muscle enzymes	High (up to 50 times normal)	High (up to 50 times normal)	High (up to 50 times normal) or normal	High	High (up to 10 times normal) or normal
Muscle biopsy findings	Primary inflammation with the CD8/MHC-1 complex and no vacuoles	Ubiquitous MHC-1 expression but no CD8 positive infiltrates or vacuoles	Perifascicular, perimyseal or perivascular infiltrates; perifascicular atrophy	Perifascicular, perimyseal or perivascular infiltrates; perifascicular atrophy	Non-specific or diagnostic for DM (sub clinical myopathy)
Rash or calcinosis	Absent	Absent	Present	Not detected	Present

Dalakas proposed initial criteria in 1991 (1), and revised it in 2003 (2). It is very specific and sensitive and has been found to have best inter-rater reliability (13, 14). Experts have reclassified IIM based purely on their histopathological features (15).

Neither of the above mentioned criteria took into account sIBM. The Griggs criteria were proposed in 1995 and were implemented as a diagnostic guide for sIBM. These relied more on the histopathological features of the disease. Recent studies underscore the importance of the clinical features as the major determining factor in diagnosing sIBM (16). Subsequently, the European neuromuscular center (ENMC) at its 188th international workshop introduced a comprehensive set of measures that addressed both the clinical and pathophysiological manifestations of the disease (17) (Table 2). Since these criteria are fairly recent, long-term studies are required to assess their utility. A recent study proposed the ENMC criteria as being effective in diagnosing with a sensitivity of 84% (18).

**Table 2:** **Revised ENMC criteria (2011) for diagnosis of sIBM (17)**.

	Clinicopathologically defined IBM	Clinically defined IBM	Probable IBM
Duration of symptoms	>12 months	>12 months	>12 months
Age at onset	>45 years	>45 years	>45 years
Pattern of weakness	Knee extension weakness > hip flexion weakness and/or finger flexion weakness > shoulder abduction weakness	Knee extension weakness > hip flexion weakness and finger flexion weakness > shoulder abduction weakness	Knee extension weakness > hip flexion weakness or finger flexion weakness > shoulder abduction weakness
Pathological features	All of endomyseal inflammatory infiltrate up-regulation of MHC-1 rimmed vacuoles protein accumulation or 15–18 nm filaments	One or more, but not all, of endomyseal inflammatory infiltrate up-regulation of MHC-1 rimmed vacuoles protein accumulation or 15–18 nm filaments	One or more, but not all, of endomyseal inflammatory infiltrate up-regulation of MHC-1 rimmed vacuoles protein accumulation or 15–18 nm filaments
Serum CK	Serum CK No greater than 15 times ULN^a^	No greater than 15 times ULN^a^	No greater than 15 times ULN^a^

*^a^Upper limit of normal*.

Idiopathic inflammatory myopathies are commonly a part of overlap myopathy (OM) and/or antisynthetase syndrome. OM are myositis associated with another defined collagen vascular disease, and a presence of “overlap autoantibodies” including MSA or myositis-associated autoantibodies (MAA). Antisynthetase syndrome is a constellation of a usually acute disease with antisynthetase antibodies, fever, interstitial lung disease (ILD) (~80%), mechanic’s hands (~70%), Raynaud’s phenomenon (60%), and polyarthritis (60%) sometimes with erosions. These patients may have clinical/pathological features of DM, PM, or NAM, but sometimes clinically evident myositis can be missing (14). Cancer-associated myositis is also considered in some literature as a separate entity that can be clinically or pathologically classified as DM, PM, or NAM. This accounts for almost half of all non-inclusion body IIM after age 65 years, but <10% in younger populations (2). A recent meta analysis involving patients with either PM or DM showed a strong association between PM/DM and malignancy (19).

## Epidemiology

As IIM are rare, few epidemiologic studies have been published. There is a growing need for accurate and reliable epidemiological studies. Between 1947 and the 1990s, the reported annual incidence of IIM from studies using older diagnostic criteria ranged 0.4–1.0 cases per 100,000 (2, 20–24). Recent study in U.S. showed that the incidence and prevalence of DM is 1.4 and 5.8 cases per 100,000 persons, respectively; with female preponderance and a higher prevalence among older age group (25). PM age- and gender-adjusted incidence was 3.8 and the prevalence is 9.7 per 100,000 people. In the opinion of the authors and other experts, PM is over-diagnosed as not all studies were based on diagnostic muscle biopsies (25).

In a retrospective study, NAM represented 19% of the IIM, while DM and non-specific myositis accounted for 36 and 39%, respectively. This study excluded sIBM (26). A Mayo Clinic study showed PM as the most common clinical phenotype (27). The incidence of DM and PM increased with advancing age and reached a peak at age 50–59 years (28). However, sIBM is still considered to be the most frequent acquired myopathy after 50 years of age. The prevalence of sIBM in Australia is 9.3 per million people in the general population and 51.3 in people over 50 years, with a male preponderance (29, 30).

## Clinical Features

### Dermatomyositis

*Dermatomyositis (DM)* typically presents as an acute or insidiously progressive proximal weakness that is accompanied or preceded by a characteristic skin rash (31–33). Patients complain of difficulty getting up from a chair, climbing stairs, lifting things, and combing hair. It is usually painless, but pain can be a significant feature with acute disease and subcutaneous calcifications. Some patients develop dyspnea related to ILD or ventilatory muscle weakness, dysphagia due to esophageal or pharyngeal involvement, congestive heart failure or arrhythmia from myocarditis, and gastrointestinal bleeding due to vasculopathy of the gut.

**Figure 1:**
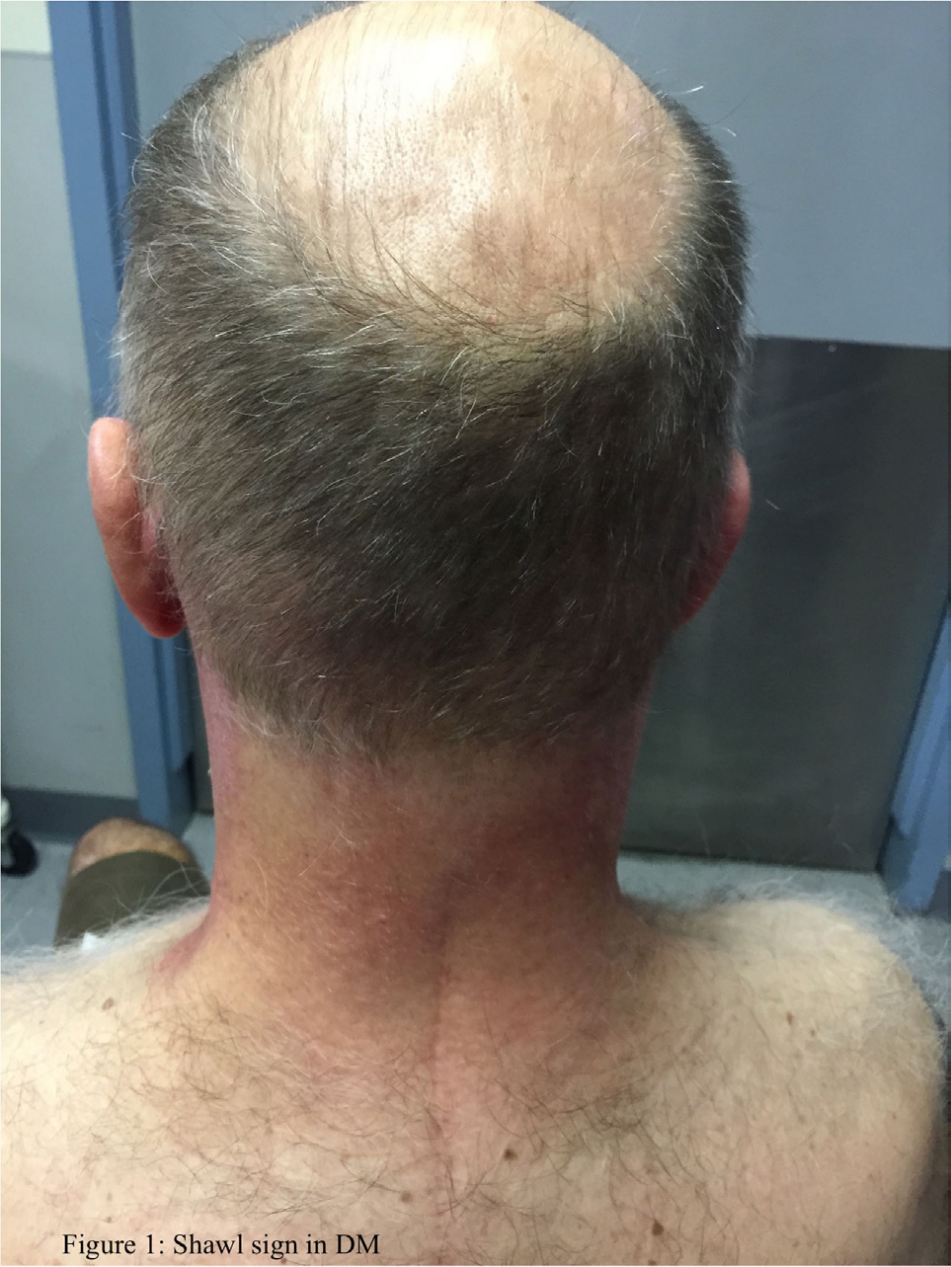
**Shawl sign in DM**.

**Figure 2:**
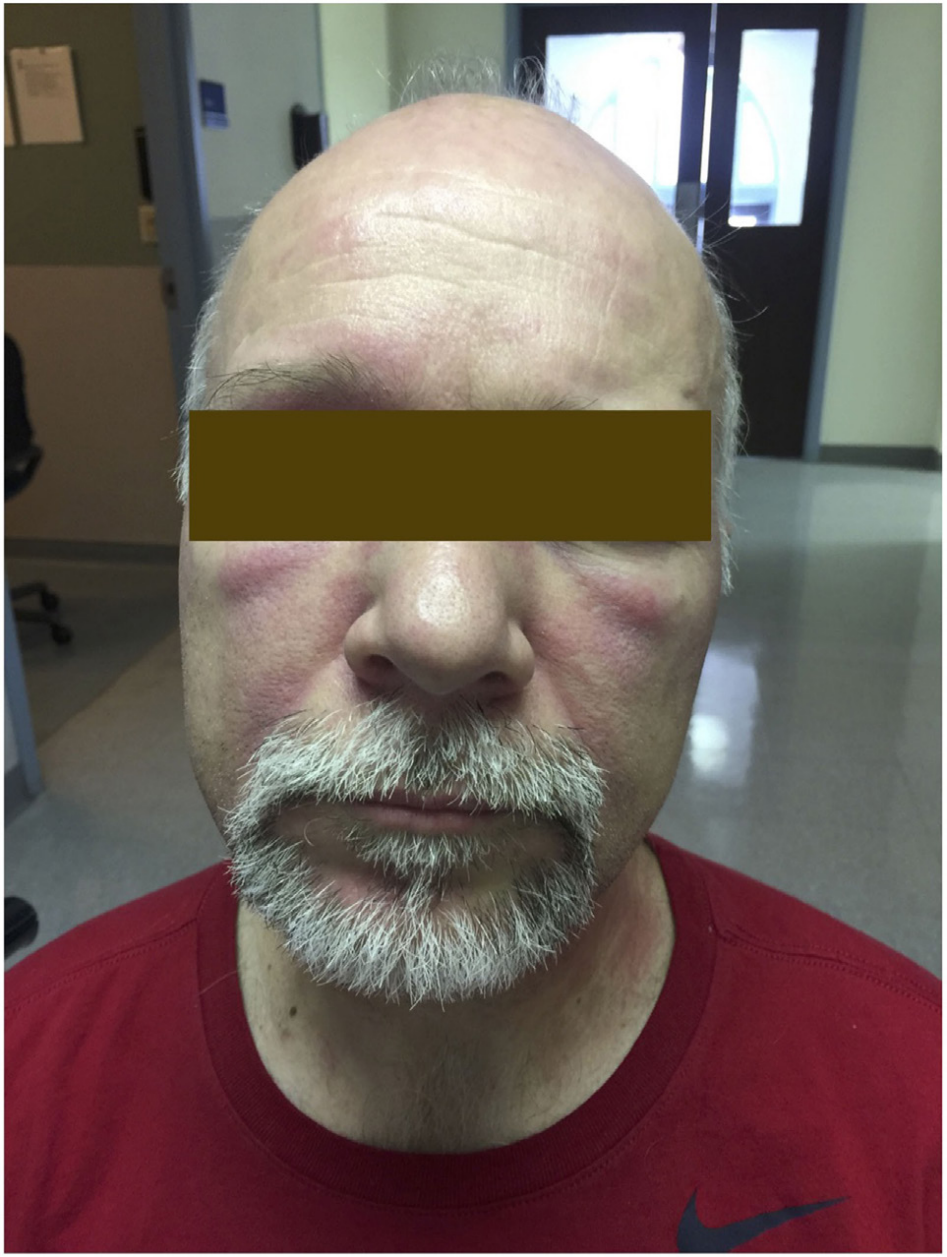
**Heliotrope rash of dermatomyositis**.

**Figure 3:**
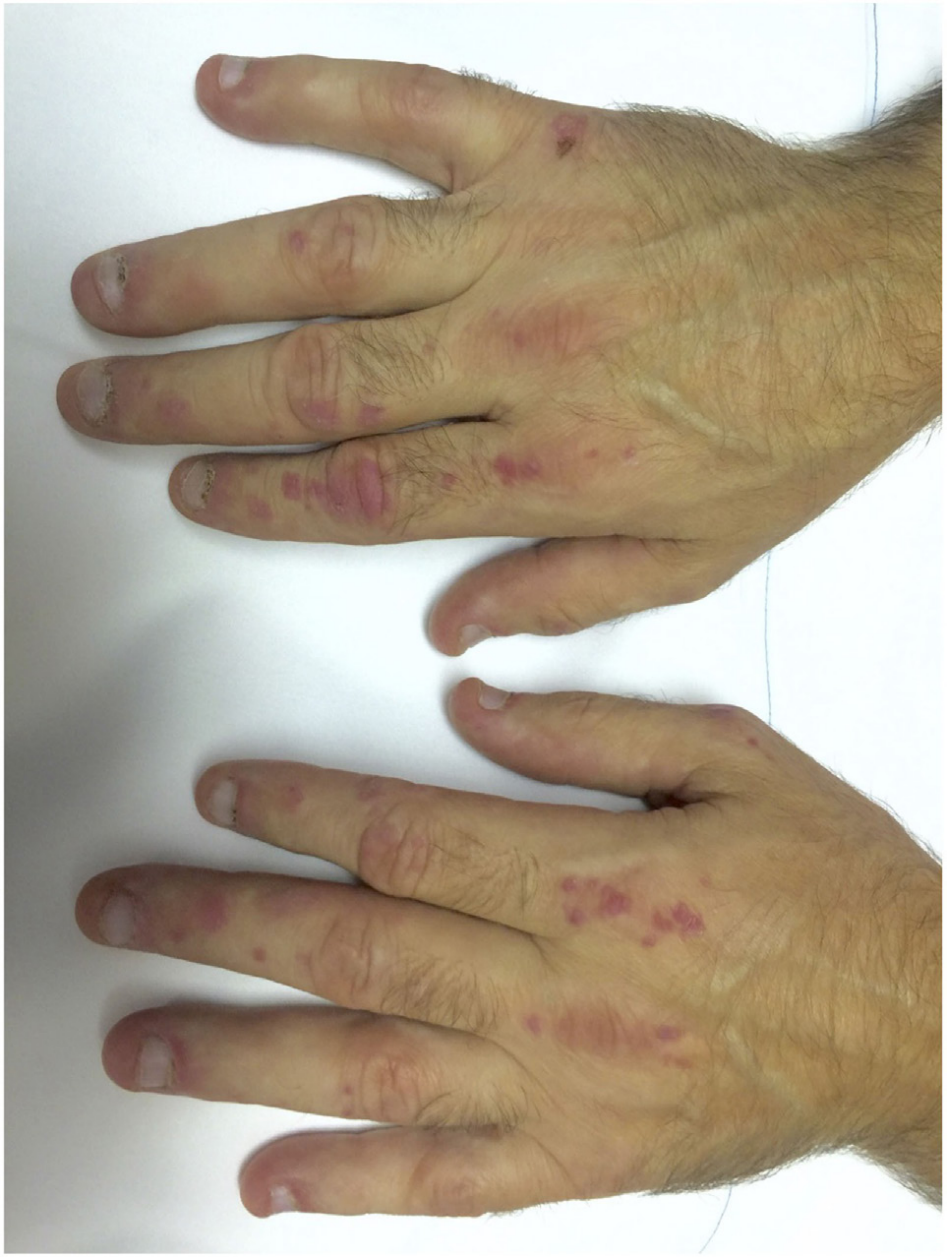
**Gottron’s papules in a case of dermatomyositis**.

**Figure 4:**
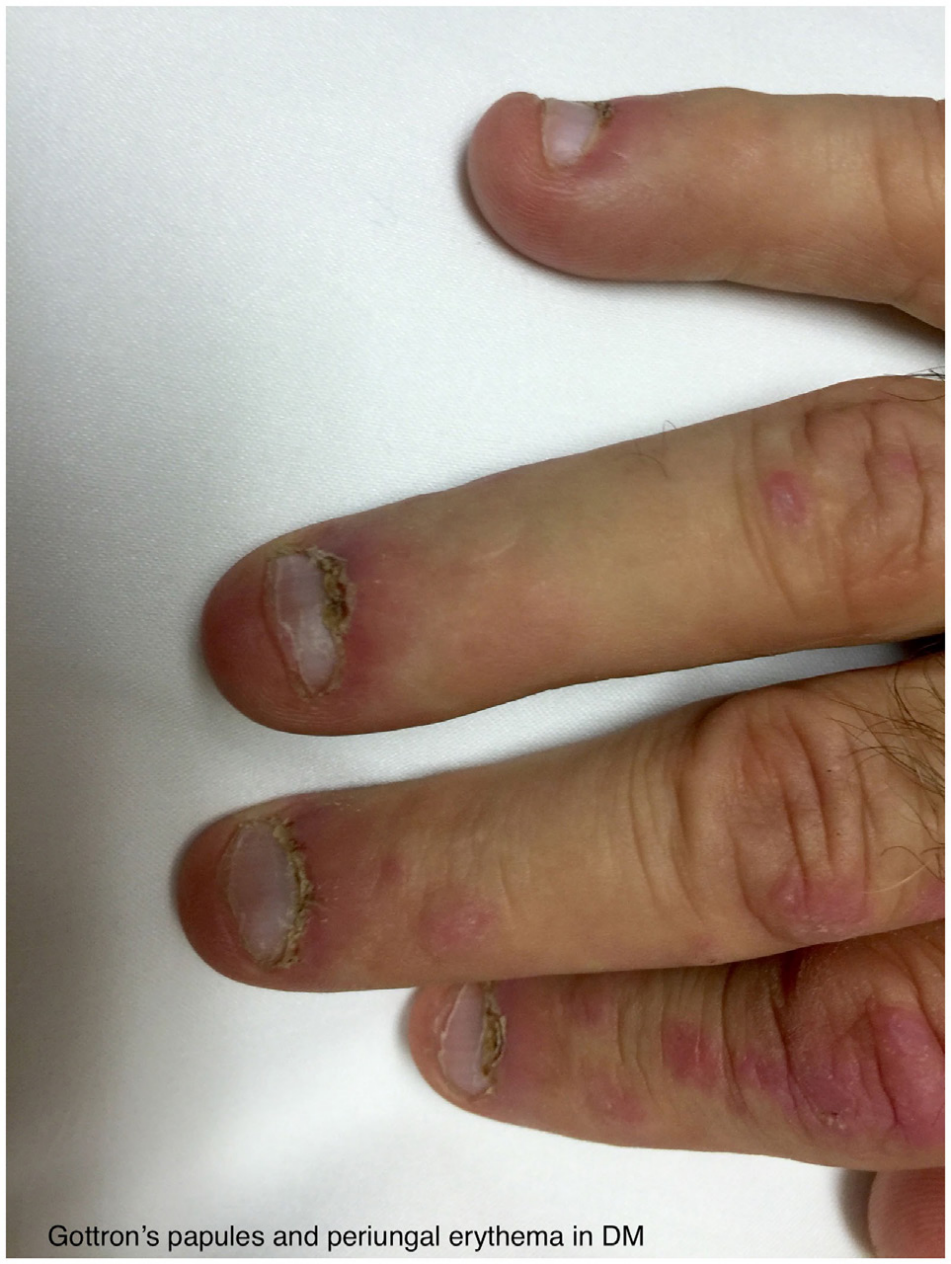
**Gottron’s papules and periungal erythema in DM**.

The typical skin rashes include: erythematous, photosensitive rash on the neck, back, and shoulders (shawl sign) (Figure 1); Malar and facial erythema along with purplish discoloration of eyelids (heliotrope rash) that is often associated with periorbital edema (Figure 2); and erythematous lichenoid papular scaly rash over the knuckles (Gottron’s papules) (Figure 3). Less commonly, rash may affect the anterior chest (V-sign) and the volar aspect of hands (inverse Gottron’s papules). Other skin manifestations include dilated capillary loops at the nail beds with periungual telangiectasias (Figure 4) and thickened, cracked skin on the dorsal and ventral surfaces of the hands (mechanic’s hands) in which case it is more often than not associated with the “antisynthetase syndrome.”

Dermatomyositis may present by itself or be a part of other syndromes, e.g., antisynthetase syndrome and overlap syndromes.

Antisynthetase syndrome is the constellation of Raynaud’s phenomenon, arthritis, and ILD. It presents with mechanic’s hands (as mentioned above). It is characterized by the presence of antibodies to aminoacyl transfer ribonucleic acid (RNA) synthetases (34).

Overlap syndrome is an entity that satisfies criteria of at least two connective tissue diseases most notably systemic sclerosis, PM/DM, Sjogrens syndrome, and SLE. Some retrospective studies have showed presence and prevalence of IIM in combination with other autoimmune diseases (35, 36).

Amyopathic DM presents with cutaneous manifestations without the muscle involvement (37), while adermatopathic DM presents with isolated myositis and has pathological features of DM on muscle biopsy. Juvenile dermatomyositis (JDM) affects children younger than 18 years of age; commonly presents after a febrile episode and skin rash. Multisystem involvement is common in JDM and is associated with calcinosis cutis and vasculopathy affecting the gastrointestinal tract (38, 39). The presence of calcinosis cutis suggests active disease in JDM and may be associated with delay to diagnosis and treatment (40). Classically, calcinosis is found at the subcutaneous level, but it may be seen intramuscularly.

### Polymyositis

*Polymyositis (PM)* is a rare entity and an exclusionary diagnosis. It presents with muscular and extra muscular organ involvement similar to DM, without a rash (6, 41, 42).

It usually manifests in adults, more commonly in women, over the age of 20 years (2, 3, 32). Unlike DM, PM is usually not seen in childhood. It presents typically with progressive neck flexor and symmetric proximal limb muscle weakness, which develops over weeks to months. Myalgias and tenderness are common complaints. Dysphagia occurs in one-third of patients. The most common extra muscular involvement is ILD and myocarditis.

### Necrotizing Autoimmune Myopathy

*Necrotizing autoimmune myopathy (NAM)* presents in adults with a sub acute, progressive proximal muscle weakness without a rash. Weakness generally develops more rapidly than PM, and is markedly severe (26). There may be associated myalgias and dysphagia. CK is usually higher than seen with other IIM. NAM is thought to be immune mediated with a trigger such as drugs (43–46). NAM has several variants including paraneoplastic-necrotizing myopathy, which is a severe and rapidly progressive disease that affects adults over the age of 40. Necrotizing myopathy with pipestem capillaries affects a similar age group and is associated with sub-acute weakness, brain infarction due to vasculitis, or connective tissue disease. Signal recognition particle (SRP) autoantibodies affect younger NAM patients, women more than men. It results in fulminant weakness and congestive heart failure. Statin-induced autoimmune NAM (SANAM) affects individuals between 46 and 89 years of age. The onset may be delayed up to 10 years following statin initiation and may occur several months after statin discontinuation (24, 45).

### Sporadic Inclusion Body Myositis

**Figure 5:**
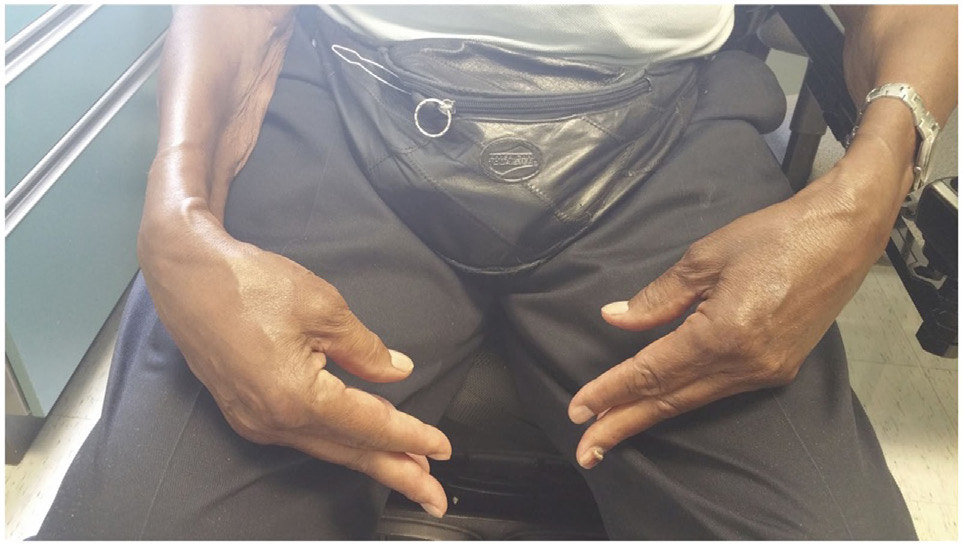
**Wasting of the small muscles of the hands and forearm in sIBM**.

Sporadic inclusion body myositis (sIBM) presents in-patients over the age of 40 years, with a male to female ratio of 3:1 (47, 48). It presents in an insidious fashion with progression over several years. Unlike other IIM, it is unique in that it involves both the proximal and distal musculature in a symmetric or an asymmetric fashion. The weakness starts in flexor forearm muscles in two-thirds of patients along with significant atrophy, particularly the deep finger flexors (Figure 5). The quadriceps and anterior tibial muscles are also affected early in IBM leading to tripping and falling. Dysphagia is very common in sIBM and may be the presenting feature (1, 49). In contrast to PM and DM, mild facial weakness is common (10). The Griggs criteria (50) address several clinical, laboratory, and histopathological features: duration of illness longer than 6 months; age at onset older than 30 years; weakness of proximal and distal muscles of the upper and lower extremities and either finger flexor weakness, wrist flexor greater than wrist extensor weakness, or quadriceps weakness; serum CK level less than 12 times normal; and muscle biopsy with evidence of invasion of non-necrotic fibers by mononuclear cells, vacuolated muscle fibers, and intracellular amyloid deposits or 15- to 18-nm tubulofilaments on muscle biopsy.

## Diagnostic Evaluations

### Laboratory Studies

#### Serum Creatine Kinase

*Serum Creatine Kinase (CK) level* is the most sensitive measure but does not correlate with the severity of the symptoms; it might improve with treatment. In DM, 70–80% will have up to 50-fold levels, while 20% of DM patients will have normal CK levels (51). These patients might rarely have isolated elevated aldolase levels. In PM the elevated levels range 5 to 50-fold above normal. CK levels in NAM can be extremely high and reaching 100-fold. In sIBM, CK levels can be normal or only mildly elevated, less than 10 times the upper limits of normal.

#### Other Muscle Enzymes

*Other muscle enzymes* include lactate dehydrogenase (LDH), aspartate aminotransferase (AST), alanine aminotransferase (ALT), and aldolase levels. They are less sensitive, and may not be elevated. Aldolase can be selectively high in myositis with perimysial pathology (15).

#### Erythrocyte Sedimentation Rate

*Erythrocyte sedimentation rate (ESR)* is not a reliable indicator as it is usually normal or only mildly elevated. The same holds true for C reactive protein (CRP).

#### Connective Tissue Disease Autoantibodies

The presence of these antibodies suggests that the myopathy may be secondary to a connective tissue disease (overlap syndrome). However, it does not necessarily establish a connective tissue disease diagnosis. Antinuclear antibodies (ANA) are detected in 24–60% of DM, 16–40% of PM, and in as many as 20% of patients with IBM (31, 47, 51, 52). Anti-Ro(SSA) and Anti-La(SSB), anti-Smith, anti-RNP, anti-Scl70, and anti-centromere antibodies should be also checked.

#### Myositis Specific Antibodies

Myositis specific antibodies have a controversial pathogenic role. They may occasionally define the clinical phenotype, and offer a prognosis for a subset of patients; most are predictors of poor treatment response (53–56).

The MSA include: (1) Cytoplasmic antibodies directed against Mi-2 and Mas antigens. (2) Antibodies targeting translational proteins such as tRNA synthetases, anti (SRP), transcriptional intermediary factor-1 gamma (TIF-1; anti-155/140 Ab), and the melanoma differentiation-associated gene-5 (MDA5; anti-CADM140 Ab).

Jo-1 autoantibody is the most common tRNA synthetase antibody (up to 20% of IIM). The other antisynthetases (PL-7, PL-12, EJ, KS, OJ, Ha, and Zo) occur in <5% of IIM (57, 58). They all lead to a similar phenotype with ILD, arthritis, Raynaud’s phenomenon, and mechanics hands (59).

Antibody to nuclear matrix protein NXP2 (or MJ antibody) is one of the most common MSA in JDM, but occur in <2% of adult DM cases with up to 50% having an associated malignancy.

The anti-155/140 autoantibodies target TIF-1 and are strongly associated with malignancy in adults (89% specificity) (60, 61). However, in JDM patients, they are associated with calcinosis rather than cancer (62).

The anti-MDA5 antibodies mostly described in Asians is associated with amyopathic DM and aggressive ILD (63).

Antibodies to Mi-2, a 240-kDa nuclear helicase, are found in 15–30% of DM patients and associated with a favorable prognosis (64, 65) and suggestions of environmental trigger in adult DM (66).

The anti-SRP antibodies are associated with NAM or maybe non-specifically positive (67). Patients present with acute and severe proximal weakness, dilated cardiomyopathy, ILD, and are often steroid resistant (68).

Anti 3-hydroxy-3-methylglutaryl-coenzyme A reductase (HMG-CoAR) antibodies (200/100 autoantibodies) have been described in patients with NAM and statin use (69).

### Screening for Malignancy

Malignancies are associated with DM (25%), PM (10%), and have been noted with NAM. Most are diagnosed within 1 year, but can take up to 5 years (7, 70). Breast and ovarian cancers are common in women, whereas lung and prostate cancers predominate in men. Other malignancies include lymphoma, colon, pancreatic, and bladder cancer (71).

Data on cancer screening are limited, and there are no guidelines. The European federation of Neurological societies recommended that DM patients have computed tomography (CT) of the chest/abdomen, pelvic ultrasound and mammography in women, ultrasound of testes in men, and colonoscopy in men and women over 50. If primary screening is negative, repeat screening is recommended after 3–6 months; thereafter screening is recommended every 6 months for 4 years (72). Periodic tumor markers (prostate specific antigen PSA, CA-125, CA19-9, CEA, and AFP) can also be checked.

### Electrophysiology

Nerve conduction study (NCV) should be done to rule out a neuropathic process. Low compound muscle action potentials (CMAP) amplitudes may not necessarily indicate a neuropathic process, as it may reflect muscle atrophy and fibrosis.

Electromyography (EMG) must be done on one side of the body, so that the muscle biopsy is done on the contralateral side. Almost always, it shows “irritable myopathic process” (73) characterized by:

(1) Increased insertional and spontaneous activity with fibrillation potentials, positive sharp waves. Muscle fibrosis in advanced cases may lead to reduced insertional activity. Worsening strength with no abnormal spontaneous activity suggests steroid induced myopathy. Pseudomyotonic discharges can be seen in NAM.
(2) Polyphasic motor unit action potentials (MUAPs) of short duration and low amplitude, with early recruitment patterns. In sIBM, there are long-duration, large-amplitude, and polyphasic MUAPs secondary to the chronicity of the disease as opposed to a neurogenic process (74).

### Skeletal Muscle Imaging

Magnetic resonance imaging (MRI) with T1 weighted, T2 weighted, fat suppression, and short tau inversion recovery (STIR) sequences have become a diagnostic modality routinely used to confirm the diagnosis, identify a muscle site for biopsy, and monitor treatment response (75–77). It is commonly used to diagnose children with JDM as it can spare the invasive testing; EMG or muscle biopsy (78). It also may help to identify subclinical involvement of muscles, which may point to another disease such as a muscular dystrophy.

In early stages, T2-weighted images and STIR sequences show patchy or diffuse increased signal in proximal muscles suggesting edema. In DM, the connective tissue septa and muscle fascia may be involved. These signal changes correlate with the degree of muscle inflammation and disease activity. After few months, T1-weighted images may show muscle atrophy, fatty transformation, and chronic muscle damage. This usually selectively involves the hamstrings with relative sparing of the adductor and obturator muscles (79).

## Histopathology and Pathogenesis

Muscle biopsy is the gold standard to make the diagnosis. It also helps to differentiate inflammatory myopathy from some muscular dystrophies that can mimic it clinically. To maximize the yield a moderately weak muscle should be chosen for biopsy. A mildly weak muscle biopsy may not be as sensitive, and a severely weak muscle may show fibrosis. MRI can help identify the affected muscles. Vastus medialis is commonly chosen; care should be taken to avoid needle EMG at the site to be biopsied.

### Dermatomyositis and Juvenile Dermatomyositis

**Figure 6:**
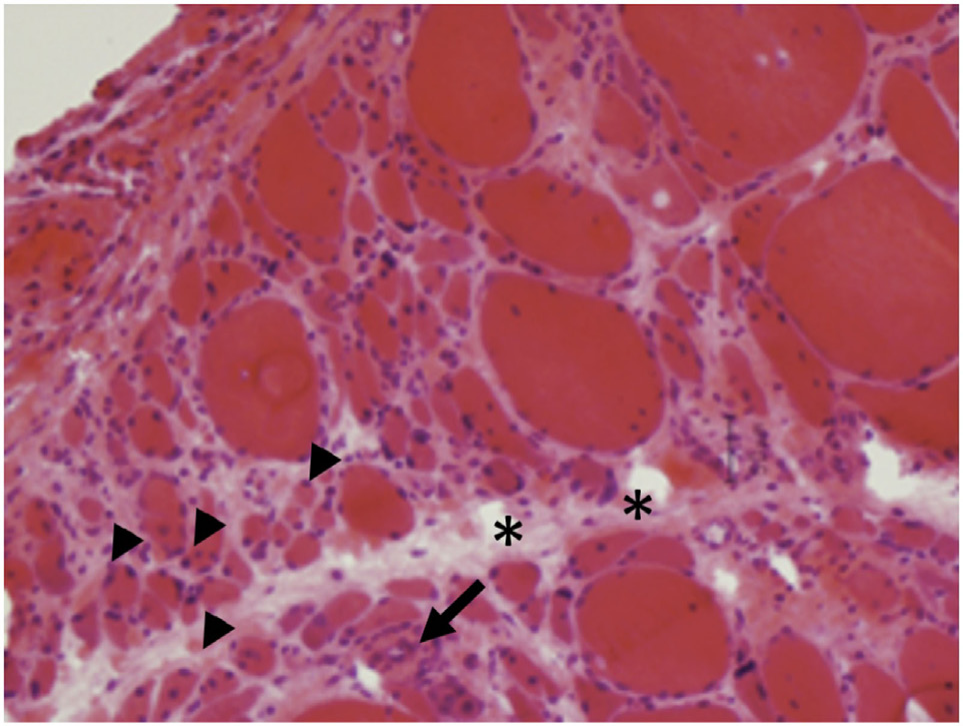
**Perifascicular atrophy (arrowheads) with increased endomysial connective tissue (asterisks) and inflammatory infiltrates (arrows) are characteristics of dermatomyositis (A, H&E, 40×)**.

**Figure 7:**
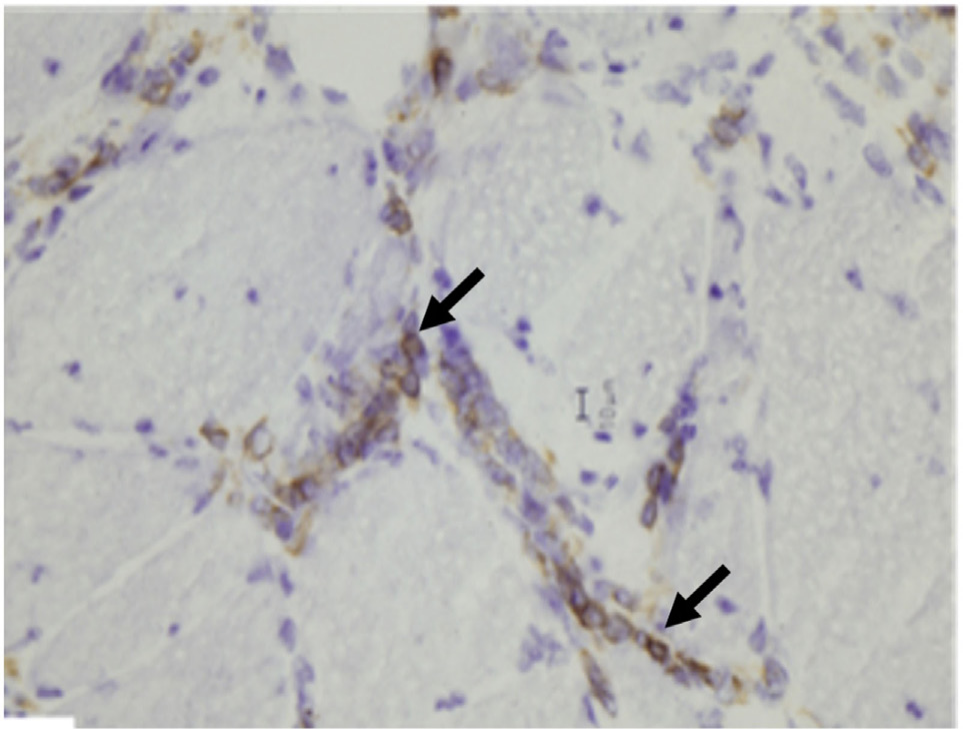
**Aggregates of B lymphocytes (arrows) positive for CD20 immunohistochemical stain are found in dermatomyositis (B, CD20 immunostatin, 40×)**.

*Dermatomyositis and JDM* are humoral-mediated vasculopathies of the small vessels in muscle tissue. This may be caused by overexpression of type 1 interferons α/β (INF-1) by plasmacytoid dendritic cells (DCs) (80) as well as increased major histocompatibility-I (MHC-I) and immunoglobulin gene transcript (81). The DCs produce type 1 interferon in response to viral nucleic acid that binds to their toll-like receptors (TLR-7 and TLR-9) (82). The activated TLR leads to generation of cytokines and chemokines including TNF-α, IL-4, IL-6, IL-15, and IL-17. Cytokines lead to cell migration and mononuclear cell infiltration in muscle fibers. The cell infiltration consists of B cells and CD4^+^T cells in the perimysial and perivascular area, and plasmacytoid DCs in perifascicular areas (4–6, 83–85) (Figure 6). On immunohistochemical stain, aggregates of B lymphocytes positive for CD20 are found (Figure 7).

The earliest histological abnormality is the deposition of the C5b-9 membrane attack complex (MAC) around the microvasculature (86, 87). This will lead to abnormalities in both perimysial intermediate-sized vessels and endomysial capillaries within regions of perifascicular myofibers. Chronic immune vascular damage may cause ischemia, myofiber atrophy, and capillary damage in “watershed” regions (88). As a result, muscle biopsies demonstrate perifascicular atrophy, often without an inflammatory infiltrate. On electron microscopy (EM), the earliest recognized changes are tubuloreticular inclusions in the intramuscular arterioles and capillaries (89).

The typical histological findings on skin biopsy are vacuolar interface dermatitis with vacuolar changes of the epidermal basal layer, apoptosis, necrotic keratinocytes, and perivascular lymphocytic infiltrate and mucin deposition in the dermis (90, 91).

### Polymyositis

*Polymyositis* is a cell-mediated cytotoxic immune response. CD8^+^ cytotoxic T cells and macrophages clonally expand and infiltrate the endomysium. They surround and invade non-necrotic muscle fiber cells expressing MHC class I, attack myocytes through the perforin pathway, causing muscle fiber necrosis and regeneration. (6, 92, 93) The microvasculature is not involved. The sarcoplasmic reticular pattern of internal MHC-1 reactivity is characteristic (94). There are abundant myeloid DCs that surround non-necrotic fibers and act as antigen presenting cells (APCs) (95). There is an increased immunoglobulin gene expression, with no deposition in the muscle blood vessels (96).

**Figure 8:**
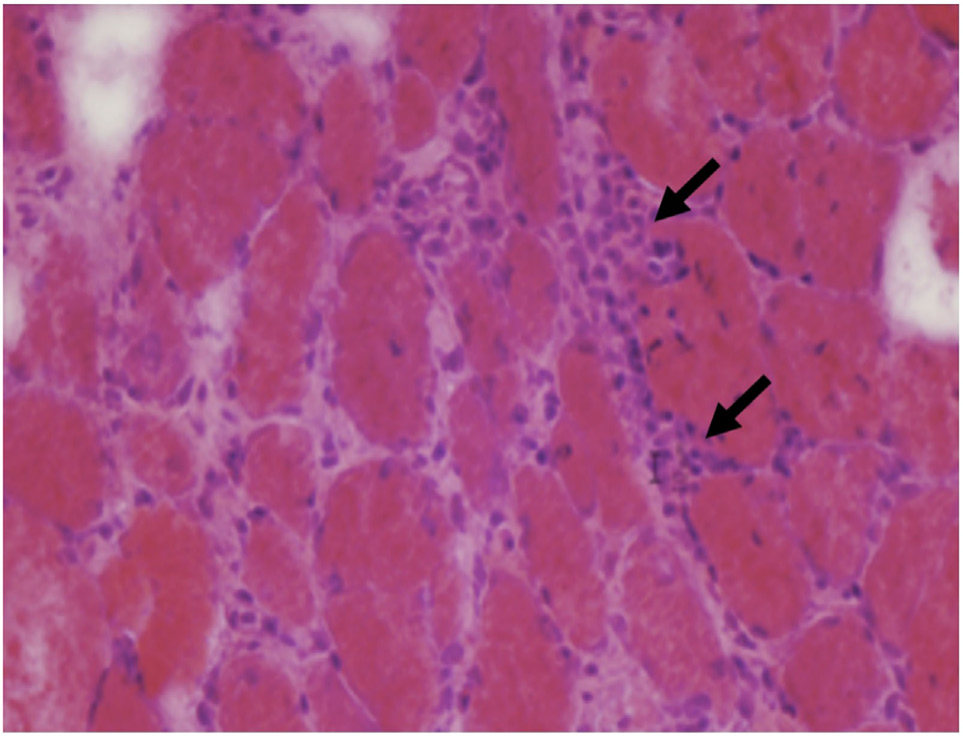
**Muscle biopsy from a patient with polymyositis showing endomysial inflammatory cells (arrows) and variations of fiber size without a specific pattern (C, H&E 40×)**.

**Figure 9:**
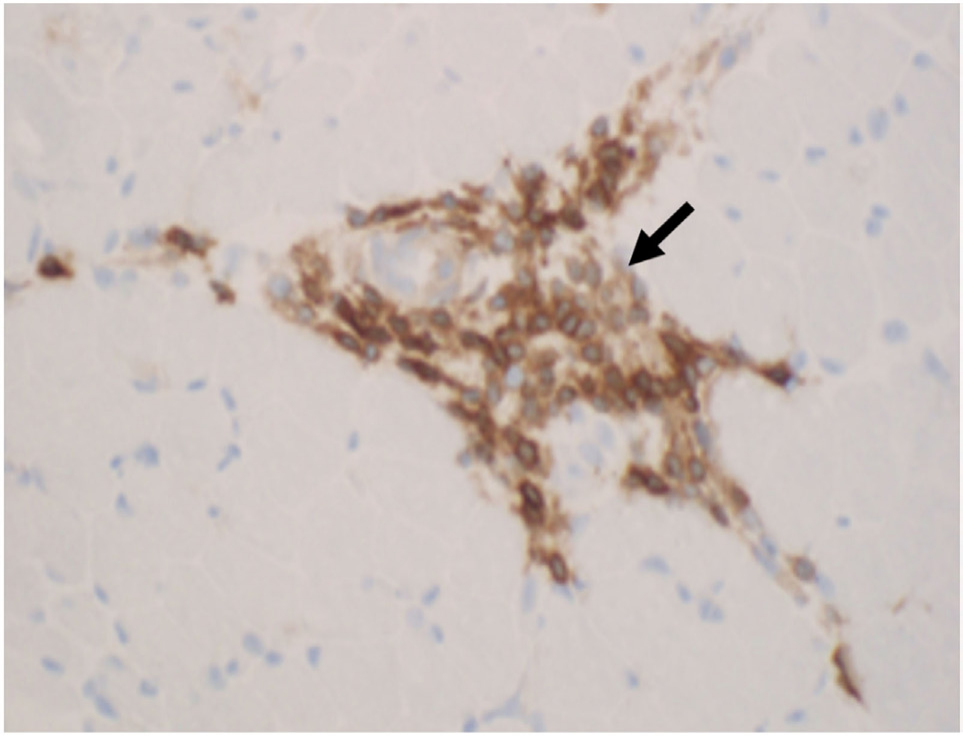
**The inflammatory cells (arrows) in polymyositis are mostly T cells that are highlighted by CD3 immunohistochemistry (D, CD3 immunostatin, 40×)**.

The main muscle biopsy features are fiber size variability, cellular invasion of non-necrotic muscle fibers expressing MHC-1 antigens, and scattered necrotic and regenerating fibers (Figures 8 and 9).

### Sporadic Inclusion Body Myositis

*Sporadic inclusion body myositis* has inflammatory and degenerative mechanisms. It remains controversial if the inflammatory mechanisms are cause or consequence of the degeneration.

**Figure 10:**
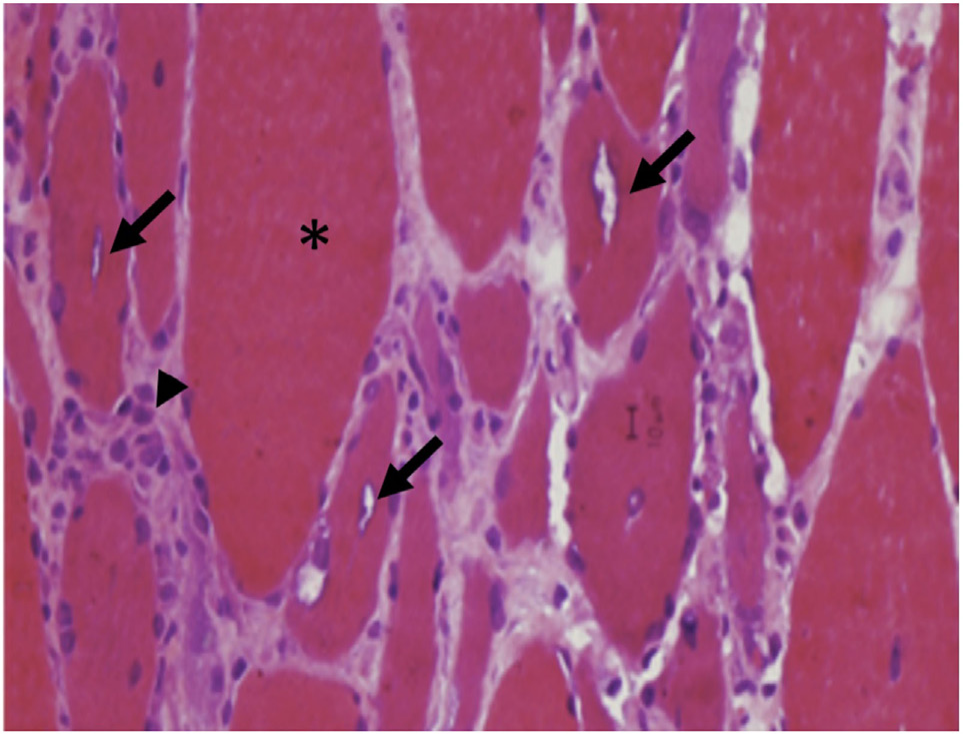
**Atrophic and hypertrophic fibers (asterisk) with internal, rimmed vacuoles (arrows) are typical findings in inclusion body myositis**.

**Figure 11:**
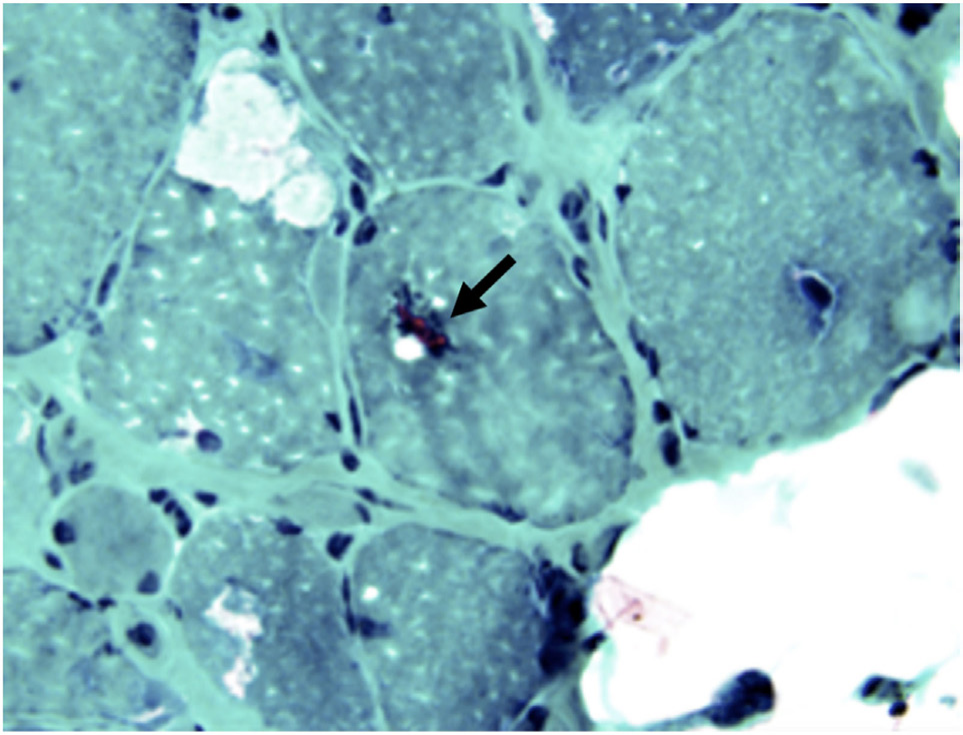
**Rimmed vacuoles (arrow) in sIBM can be highlighted by modified trichrome stain (F, Modified trichrome stain, 40×)**.

The inflammatory process is similar to PM with an invasion of non-necrotic fibers by macrophages and cytotoxic CD8^+^ T cells. The degenerative process is characterized by rimmed vacuoles that can be highlighted on modified trichrome stain, and sometimes, ragged red fibers with mitochondrial excess, eosinophilic inclusions, and cytochrome oxidase negative fibers (50, 97, 98) (Figures 10 and 11). On EM, nuclear and cytoplasmic inclusions are detected. Macroautophagic processing has been attributed to the accumulation of aberrant proteins, such as congophilic intracellular β-amyloid deposits, presenilin 1, apolipoprotein E, γ-tubulin, α-synucline, and phosphorylated tau proteins (99). They accumulate as 12–16 nm filamentous masses, reported to be identical to the paired helical filaments found in the brains of Alzheimer’s disease (100).

Inducible nitric oxide synthase under pro-inflammatory conditions is upregulated and causes myofiber death (101).

### Necrotizing Autoimmune Myopathy

*Necrotizing autoimmune myopathy* is thought to be macrophage-mediated immune response (43, 44). Other findings suggest an antibody-mediated mechanism in some subtypes (anti-SRP and anti-HMG-CoAR) (45, 69). The major findings on biopsy are scattered necrotic muscle fibers (46), surrounded by sparse inflammatory cells, predominately lymphocytes and occasionally some CD4^+^ and CD8^+^ T cells (102). These are detected through immunostaining (CD3, CD68). The overexpression of MHC-I in necrotic and regenerating fibers is variable (103, 104). NAM shares some features with DM of MAC deposition on micro vessels, but without perivascular inflammation, perifascicular atrophy, or tubuloreticular inclusion in endothelium. A variety of distinctive findings occur in specific subtypes of NAM. Demonstration of thickened basement membranes and enlarged pipestem capillaries of normal number is diagnostic of NAM with pipestem capillaries (43).

## Treatment

The main goals of IIM therapy are to restore muscle strength, limit/eliminate the inflammation, and prevent other organs damage. It is ideal if the treatment involved a multidisciplinary approach; neurology, rheumatology, dermatology, pulmonary, physical occupational, and speech therapy.

There are only a few published prospective, double-blind, placebo-controlled trials for the treatment of IIM.

In general, NAM is more resistant to immunosuppressive therapy than DM and PM, particularly if there is an underlying malignancy or a statin trigger. The vast majority of sIBM patients are poorly responsive to immunotherapy.

### Initial treatment Approach in Adults

#### Corticosteroids

*Corticosteroids* (high-dose) is the first line of treatment for adult onset DM, PM, and NAM (51). Its effect has never been formally proved in a prospective double-blind study, but the therapy is based on early reports suggesting a positive effect of corticosteroids on muscle strength (105). The initial prednisone dose is 0.5–1 mg/kg/day (60–100 mg once daily). Depending on patient response, taper usually take place after 4–6 weeks or when strength improvement reaches a plateau. Multiple tapering regimens have been used; one of the tapering regimens is 10 mg q2weeks to reach 30 mg/day, then 5 mg q2weeks to reach 20 mg/day, then 2.5 mg q2weeks until taper is completed or until reaching the lowest dose that will keep the patient in sustained remission (12). Others use taper to alternate day dosing over 2–3 months. However, some patients may not tolerate the swings, especially diabetics (74). Monthly 4-day course of 40 mg dexamethasone as an oral pulse therapy displayed a comparable efficacy as daily prednisone, but significantly less side effects. (106) Serum CK levels should be monitored, but adjustments of treatment should be based on objective clinical examination.

No response after an adequate trial of high dose prednisone should raise suspicion of alternative diagnoses like sIBM or inflammatory muscular dystrophy. Repeat muscle biopsy should be considered. Increasing weakness after an initial response may be due to a relapse or corticosteroids related myopathy. Relapse is more common to happen during taper. An EMG can be helpful to differentiate, as it does not show muscle membrane irritability with steroid myopathy.

Common side effects of high-dose corticosteroids include sleep disturbances, exacerbation of mood disorders, psychosis, glaucoma, cataract, avascular necrosis, osteoporosis and pathological fractures, hypertension, and hyperglycemia. Dual energy x-ray absorptiometry (DEXA) should be obtained at baseline and yearly while patients are receiving corticosteroids. Calcium (1 g/day) and vitamin D (400–800 IU/day) are initiated for prophylaxis against steroid-induced osteoporosis (107). Bisphosphonate can be added in postmenopausal women. Proton pump inhibitors are helpful in prevention of gastrointestinal complications. Periodic ophthalmological monitoring for glaucoma and cataract is recommended. Also, fasting blood glucose and serum potassium levels should be monitored. Potassium supplementation may be required if therapy leads to hypokalemia.

The decision regarding the timing of adding a second line agent may vary according to the severity of weakness, the initial response to prednisone, relapse, and in patients with increased risk of steroid complications (diabetics, osteoporosis). In most cases, starting an immunosuppressive drug at the time of initiating steroid treatment is necessary. The usual choices are methotrexate (MTX), azathioprine, and mycophenolate mofetil (MMF). There were no trials that compared these agents head to head, therefore, there is no superiority of choosing one of them over the others.

#### Methotrexate

*Methotrexate* is an antifolate that inhibits lymphocyte proliferation. The initial dose is 7.5 mg once weekly. Folic acid 1 mg daily or leucovorin 5 mg weekly on the subsequent day are important to limit some side effects (108). The dose can be increased by 2.5 mg weekly to reach target dose of 25 mg once weekly. With higher doses MTX can be given in three divided doses 12 h apart. Therapeutic effects of oral MTX are often noticeable after 4–8 weeks. If there is no improvement after 1 month of 25 mg weekly of oral MTX, some experts switch to weekly subcutaneous MTX and increase the dose by 5 mg every week up to 60 mg weekly (74).

Gastrointestinal side effects and alopecia are common. Painful stomatitis can happen and may respond to higher dose of folic acid. MTX is an oncogenic and teratogenic drug, and women of childbearing potential should be advised to use two forms of birth control while taking it. It is also hepatotoxic, nephrotoxic, and myelotoxic. Therefore, baseline liver function tests (LFTs) and measurement of Hepatitis B virus (HBV) and Hepatitis C virus HCV antibodies should be done before initiating the treatment. LFTs, complete blood count (CBC), and creatinine should be checked monthly for the first 3 months, then every 3 months (109). Gamma glutamyltransferase (GGT) is a specific hepatic marker that can help determine whether transaminitis is due to hepatotoxicity or muscle involvement alone. MTX can rarely induce pneumonitis, which may be indistinguishable from myositis associated ILD. This side effect makes some experts hesitant to start MTX on patients with ILD or Jo-1 antibodies. Screening for lung disease with pulmonary function tests (PFT) periodically is advised.

#### Azathioprine

*Azathioprine* is an antimetabolite that blocks T-lymphocyte proliferation. The initial dose is 25–50 mg daily; with increment of 25 mg qweek to goal does of 2–3 mg/kg of ideal body weight (100–250 mg daily). The dose can be given daily or can be divided into twice daily regimen. Azathioprine has a delayed onset of response that begins in 4–8 months and peaks at 1–2 years.

Common side effects of azathioprine include nausea and loose stools. A flulike reversible systemic reaction affects 12% in the first 2 weeks of therapy (110). Thiopurine methyltransferase (TPMT) should be checked before initiating treatment. This enzyme deficiency may lead to myelosuppression if standard dosage of azathioprine was given. In heterozygous TPMT mutation, azathioprine dose should be reduced, while in homozygous TPMT mutation, azathioprine should be avoided (111, 112). Azathioprine is teratogenic and oncogenic and can also cause pancreatitis. Hepatotoxicity may develop within few months. Myelosuppression can develop within weeks, but may also take 2 years. Hence, as with MTX, LFTs CBCs and creatinine should be monitored monthly for the first 3 months, then every 3 months. Dose should be decreased if white blood count (WBC) falls below 4000/mm^3^ and held if WBC declines to 2500/mm^3^ or LFTs increases more than twice baseline. This toxicity may take few months to reverse. Some patients may tolerate a rechallenge when laboratory values return to baseline (110).

#### Mycophenolate Mofetil

*Mycophenolate mofetil (MMF)* is an inosine monophosphate dehydrogenase inhibitor that blocks purine synthesis and inhibits T- and B-cell proliferation. Patients with anti-synthetase syndrome may respond favorably to MMF. It is initiated at 500 mg twice daily and increased by 500 mg weekly to goal of 1000 mg twice daily, or sometimes 1500 mg twice daily. The dose should be decreased in patients with renal insufficiency (1000 mg total dose).

Mycophenolate mofetil is a well-tolerated drug, with some side effects at higher doses, mainly nausea and loose stools. Severe infections may happen. Patients on MMF should be monitored for leukopenia and transaminitis. Women of childbearing potential should use two forms of birth control. Pregnancy should not be planned before withdrawal from immunosuppressant for several months (113).

### Treatment Approach for Severe or Refractory Disease

Sometimes patients with IIM can be refractory to standard treatment or can have severe disease including severe cutaneous symptoms, severe muscle weakness, dysphagia, or significant weight loss. More aggressive treatment should be chosen for these groups.

#### Intravenous Methylprednisone

*Intravenous methylprednisone (IVMP)* can be used. Dose is usually 500–1000 mg daily for 3 days, followed by high-dose oral prednisone with taper.

#### Intravenous Immunoglobulin

*Intravenous immunoglobulin (IVIG)* has multiple indications. It can be used for refractory cases or as an add-on during relapses. IVIG is a good alternative to immunosuppressant agents in patients suffering from side effects or in childbearing women. There is little evidence that IVIG is effective as a monotherapy. It has a complex immunomodulatory mechanism of action including: (1) inhibition of complement activation, (2) reduction of autoantibody production and binding, (3) enhancement of antigen recognition by sensitized T cells, (4) blockade of Fc receptors, (5) downregulation of phagocytosis, and (6) inhibition of cell transmigration into the muscle (114). Dose is 2 g/kg given over 2 or 5 days once a month up to 6 months.

Serum IgA should be checked before starting treatment because IgA deficiency may lead to severe anaphylaxis caused by complexes formed between infused IgA and anti-IgA antibodies (115). In cases of IgA-deficiency, IVIG with reduced IgA levels can be given. Renal function should be checked due to the risk of IVIG induced renal failure. Flu-like symptoms including headaches, myalgias, fever, chills, nausea, and vomiting are common (up to 50%) and can be premeditated with acetaminophen and diphenhydramine. Aseptic meningitis, myocardial infarction, and stroke can also complicate IVIG administration.

#### Rituximab

*Rituximab* is a human monoclonal antibody to CD20 that act through depletion of B cells in circulation. It is effective in treating refractory IIM including SRP positive patients. It is given in two doses course, 1000 mg dose 2 weeks apart. This course can be repeated in 6–9 months. Baseline immunoglobulins, HBV and HCV antibodies, and tuberculosis screen should be done prior to starting the treatment. Recent trials have shown that rituximab is an effective choice for treatment resistant IIM, especially that in association with antisynthetase syndrome (116, 117). A recent retrospective review of charts of patients with refractory PM and DM treated with rituximab showed an objective improvement in regards to the CPK values and lung function tests. It also showed that patients with antisynthetase syndrome frequently required retreatment, and that, infections were a major limiting factor in treatment (118). Adverse effects can range from fever, chills, to bronchospasm, neutropenia, and thrombocytopenia. Severe infections can happen. Progressive multifocal leukoencephalopathy has been reported (119).

#### Cyclophosphamide

*Cyclophosphamide (CYC)* is a nitrogen mustard-alkylating agent that blocks both T- and B-cell proliferation. It is used when all other treatments fail or with severe multi-organ manifestations. This is due to the serious adverse effects including cytopenia, hemorrhagic cystitis due to metabolizing to acrolein, premature ovarian failure, and severe infections. Other major side effects are GI upset, alopecia, teratogenicity, and oncogenicity. It can be given as weekly intravenous infusion of 0.6–1 g/m^2^ after adequate oral and intravenous hydration, antiemetics, and mesna. The weekly infusions can be given up to 6 months, and sometimes 12 months. Mesna should be given with and after 4 and 8 h of CYC infusion to reduce the incidence of hemorrhagic cystitis. CYC can be also given orally as 1–2 mg/kg daily dose. Patients should maintain good hydration and frequent urination. Urinalysis and CBC are monitored every other week. A nadir of WBC that occurs 2 weeks after the IV infusion should not fall <3000/mm^3^.

#### CyclosporineA and Tacrolimus

*CyclosporineA (CSA) and tacrolimus* are calcineurin inhibitors that suppress helper T lymphocytes and block the production and secretion of interleukin-2. Pulmonary improvement was noted in patients with anti-Jo-1 and anti-SRP antibodies who were treated with tacrolimus. Both drugs should be started at a low dose and titrated slowly to 6 mg/kg/day for CSA and to 0.2 mg/kg/day for tacrolimus. The use of CSA and tacrolimus is limited because of serious adverse effects including hypertension, electrolytes imbalance, and renal insufficiency (120). Other side effects include GI upset, gum hyperplasia, hirsutism, oncogenicity, tremor, and risk of infection. Serum CSA and tacrolimus trough levels are monitored routinely to avoid renal toxicity.

#### Tumor Necrosis Factor Alpha Blockers

*Tumor necrosis factor alpha (TNF-a) blockers* like infliximab and etanercept were tried and showed steroid sparing effect (121).

### Emerging Therapies

#### Anakinra

*Anakinra* is a recombinant human IL-1 receptor antagonist used commonly for the treatment of Rheumatoid arthritis and is an emerging drug in the treatment of IIM (122, 123). A 12-month follow-up study showed the beneficial effects of Anakinra. Fifteen patients with refractory myositis were treated with Anakinra for 12 months and treatment response was gaged by the parameters set by the International Myositis Assessment and Clinical Studies (IMACS) group and the functional Index (FI) (123). A recent study was performed on 15 patients treated with Anakinra for 12 months at the end of which a beneficial clinical response was noted in seven of these subjects. Repeat muscle biopsies were also done to explore possible predictive biomarkers (124). There is still room for large-scale randomized trials to show the efficacy of anakinra in IIM.

#### Alemtuzumab

*Alemtuzumab* is a humanized monoclonal anti-CD52 antibody that causes an immediate and severe depletion in the peripheral blood lymphocytes. It has shown to be a promising drug for the treatment of sIBM. As sIBM is largely resistant to treatment with steroids and immunosuppressive agents and has a rapidly deteriorating course. In a “proof-of-principle” study on thirteen sIBM patients, Dalakas et al. showed that alemtuzumab infusions slowed down disease progression up to 6 months and improved muscle strength (125). Recently, a long-term follow-up case study on a treatment-resistant case treated with alemtuzumab showed marked improvement in muscle strength 12 weeks into a single treatment cycle with alemtuzumab and this lasted for ~3 years (126).

#### Belimumab

*Belimumab* is a human monoclonal antibody directed against B lymphocyte stimulator (BLys), which is a TNF-related cytokine implicated in B cell maturation and development. It was approved for treatment of Systemic lupus erythematosus (SLE) in March 2011 (127).

Its role is currently being studied in the management of PM DM.^1^

#### Sifalimumab

*Sifalimumab* is an anti-IFN-monoclonal antibody Neutralization of the type I IFN gene signature by sifalimumab resulted in coordinated suppression of T cell-related proteins such as soluble IL-2RA, TNF receptor 2 (TNFR2), and IL-18 (128).

### Treatment Approach to JDM

The strategy is similar to the one used for adults, except that initial prednisone dose is 2 mg/kg; maximum of 60 mg daily. MTX is the main steroid sparing agent and is added at onset 15 mg/m^2^ subcutaneously weekly. Other immunosuppressive agents that can be used are azathioprine, cyclosporine, and tacrolimus. MMF is not used routinely in children. IVIG is efficacious and safe for refractory cases. Rituximab 575–750 mg/m^2^ is recently gaining acceptance. IV CYC at 0.5–1.0 g/m^2^ can be used as a last resort.

### Management of Skin Disease

To prevent skin flairs, patients with DM and JDM should avoid UV rays; use sunscreen in addition to appropriate coverage. Topical steroids and tacrolimus have been used (129).

#### Hydroxycholoroquine

*Hydroxycholoroquine* is an antimalarial drug that is used for cutaneous manifestations in DM and JDM and is given 200 mg twice daily. Baseline electrocardiogram should be obtained to screen and monitor for QT prolongation. Also a baseline ophthalmology testing is important to rule out macular disease, and subsequent annual screening after 5 years of treatment should be done to monitor for retinal toxicity (130).

### Management of Calcinosis

Therapy of calcinosis shows lack of meaningful response. Diltiazem may produce a partial response (131). There was an improvement of calcinosis with abatacept and sodium thiosulfate – a vasodilator that chelates calcium in a case report. (132) Surgical excision is an option.

### Management of Dysphagia

Dysphagia can occur in all subtypes of myositis. A high percentage of patients with IBM are affected (133), which leads to the risk for malnutrition and aspiration pneumonia. Treatment with IVIG improves swallowing in IBM (134–136) as well as prednisone-resistant DM or PM (137).

Cricopharyngeomyotomy is used when the underlying mechanisms of dysphagia is failed relaxation of the upper esophageal sphincter. This intervention may not be beneficial with normal relaxation and other mechanisms of dysphagia, i.e., delayed swallow initiation and decreased hyolaryngeal excursion (138). Other interventions include percutaneous endoscopic gastrostomy (PEG), pharyngoesophageal dilatation, and injection of botulinum toxin (139–141).

### Treatment of Associated ILD

Most patients require adjuvant immune modulating drugs with the first-line corticosteroids (57). MMF is the favored drug. Also, cyclosporine and tacrolimus are effective as second-line agents (142). Cyclophosphamide is considered a third-line agent (143). Rituximab has recently emerged as a promising effective agent in treatment of antisynthetase syndrome, which is notoriously associated with severe ILD (116). Factors predictive of poor ILD prognosis include older age, lower values of PFTs, CT scan with a pattern of interstitial pneumonia, and treatment-refractory.

### Physical Therapy

Physical and occupation therapy are essential and along with orthotic devices if needed, help patients improve mobility, retain motor function, prevent contractures that can arise especially in JDM and may help prevent steroids side effects like weight gain, osteoporosis, and type 2 fiber atrophy. Strengthening programs twice weekly can be started as early as 2–3 weeks from the acute phase (144). With severe cases, passive range of motion exercises can be done for 3 months, until strength improve; at which point strengthening exercises are initiated. There is growing evidence for safety and beneficial effects of physiotherapy and home exercise programs in myositis (145).

A recent study demonstrated the effect of a 12-week aerobic exercise program in 10 children with chronic JDM. At the end of this longitudinal study, the subjects showed an improvement in muscle strength and function, aerobic conditioning, and a better quality of life (146).

Dastmalchi et al. showed that muscle biopsies in patients with PM/DM contained a low population of oxygen dependent type 1 fibers in comparison to healthy individuals. After 5 days a week to 12-week home exercise program, repeat muscle biopsies showed a higher frequency of oxygen-dependent type 1 fiber. Through this study, a molecular basis for the beneficial effects of exercise training was established (147).

### Treatment of sIBM

Treatment of sIBM is challenging, as the disease typically is resistant to standard immunotherapy. Prednisone is usually not effective (47) and may lead to more rapid progression (30). However, some patients may experience at least a temporary improvement. Some treat newly diagnosed patients with immunosuppression (12). Rationale is that early suppression of the inflammatory cascade may prevent downstream effects leading to muscle degeneration (148).

Studies with MTX (149), anti-T-lymphocyte globulin (150), etanercept (151), oxandrolone (152), or β-interferon (153, 154) failed to identify clinical efficiency. Although IVIG can help with dysphagia, it was not found effective for muscle strength (155).

The most encouraging study used alemtuzumab, a monoclonal antibody that deplete peripheral lymphocytes. It seemed to delay the disease progression for up to 6 months, and in some patients, the muscle strength was improved (125).

The concept that modulating the action of the Heat shock response proteins (HSR) in cells can dampen the detrimental aspects of both degeneration and inflammation has recently been tested through a phase two placebo-controlled trial. For this purpose, arimoclomol, an agent that amplifies heat shock protein expression was used. The results have been encouraging and provide a platform for further large scale trials (156).

Through a pilot trial, it has recently been suggested that TGF*b* signaling through activin receptors may be responsible for the pathological changes seen in sIBM (157). In this trial, an actRII inhibitory antibody, bimagrumab was shown to result in improvement in thigh muscle volume in patients with sIBM at 8 weeks.

An ongoing phase II trial is being conducted by Novartis to prove bimagrumab’s efficacy in treating patients with sIBM^2^.

Different studies conducted for IIM therapies are outlined in Table 3. The efficacy/side effects among different IIM are briefly mentioned.

**Table 3:** **Various treatments, modalities and corresponding studies**.

	Study	RCT	No.	Results
Dexamethasone	van de Vlekkert et al. (106)	Yes	62	Pulsed high-dose oral dexamethasone is not superior to daily prednisolone as first-line treatment of IIMs, but is a good alternative by causing substantially fewer side effects
Intravenous methylprednisolone (IVMP)	Huang (158)	No	24	Most patients with JDM whose disease followed a monocyclic IVMP course achieved normal muscle enzymes and had improved muscle strength faster
	Bolosiu et al. (159)	No		Improvement was noted in the muscle limb scores, CRP and CK levels in patients treated with methylprednisolone pulse therapy, with persistence of some cutaneous features of DM
Intravenous methylprednisolone (IVMP) and methotrexate (MTX)	Al-Mayouf et al. (160)	No	12	MTX and IVMP are a useful combination in the early treatment of severe JDMS
Methotrexate (MTX)	Sokoloff et al. (161)	No		MTX therapy is a first line therapy in case of failure of steroid therapy
	Metzger et al. (162)	No		MTX therapy allowed for a decrease in steroid dosage
	Giannini et al (163)	No		Addition of MTX to prednisone therapy showed improvement in CPK
	Cagnoli et al. (164)			
Azathioprine	Bunch (165) and Bunch et al. (166)	Yes	16	Azathioprine and prednisone were not different than prednisone and placebo
	Bunch (165)	No	16	Longer follow-up has shown that the group given prednisone plus azathioprine has improved more with respect to functional disability and required less prednisone for disease control
Azathioprine vs. methotrexate (MTX)	Joffe et al. (65)	No	113	Methotrexate therapy may be superior to either azathioprine or further steroid treatment alone
Methotrexate (MTX) and azathioprine combination	Villalba et al. (167)	Yes	30	Of the 15/30 patients with refractory myositis that were initially randomized to oral MTX/AZA, 8 improved with oral therapy and 1 improved with I.V. MTX during the crossover period
Methotrexate (MTX) vs. cyclosporine (CSA)	Vencovský et al. (168)	Yes	36	MTX showed insignificantly better response than patients on CSA
Cyclosporine (CSA)	Qushmaq et al. (169)	No	65	Effective therapy for resistant PM/DM and toxicity is possibly more than expected
	Maeda et al. (120)	No	14	Cyclosporine was effective against both myositis and interstitial pneumonitis
Tacrolimus	Oddis et al. (170)			Tacrolimus was beneficial in Jo 1 positive patients with ILD
Intravenous immunoglobulin (IVIG)	Dalakas et al. (171)	Yes	15	High-dose intravenous immune globulin is a safe and effective treatment for refractory DM
Plasma exchange (PLEX)	Miller et al. (172)	Yes	39	Leukapheresis and plasma exchange are no more effective than sham apheresis
Mycophenolate mofetil (MMF)	Majithia and Harisdangkul (173)	No	7	A striking clinical and laboratory response of active myositis in 6/7 patients
	Edge et al. (174)	No	12	Improvement was seen in 10 of the 12 patients with DM who had skin lesions recalcitrant to traditional therapies, most within 4–8 weeks
	Morganroth et al. (175)	No	4	Patients experienced complete normalization or improvement of pulmonary function tests and resolution of dyspnea. They were also able to reduce their prednisone doses
	Gelber et al. (176)	No	4	In all 4 patients with DM, MMF was effective, with a mean duration of treatment of 13 months, at controlling cutaneous disease activity, resulting in a decrease of the steroid dose
Rituximab	Mok et al. (177)	No	4	Four patients with active PM had resolution or significant improvement in muscle power and CK levels, at week 28
	Cooper et al. (178)	No	4	One patient with anti-Mi-2, remained disease-free for 14 months following 2 courses of rituximab. Two myositis antibody-negative patients showed clinical improvement and tolerated lower doses of corticosteroids. One patient had worsening of her disease following rituximab
	Levine (179)	No	7	Patients with DM received weekly IV rituximab ×4. All exhibited major clinical improvement (as early as 12 weeks), including rash, and forced vital capacity
	Oddis et al. (180)	Yes	200	Patients were randomized to receive either rituximab early or rituximab late, with no difference in the time to achieve improvement. 83% of adult and juvenile myositis patients with refractory disease met the definition of improvement (76 PM, 76 DM, and 48 JDM)
	Mahler et al. (181)	No	13	The median levels of CPK and LDH were significantly reduced by 93.2 and 39.8%, respectively, after 34.6 months. The median muscle strength measured by hand-held dynamometry was significantly improved by 21.5% after 24 months
Cyclophosphamide (CYC)	Kono and Gilbert (182)			CYC pulse therapy was found to be of benefit in 3 patients with refractory PM and concomitant SLE
	Yamasaki et al. (183)	No	17	IV CYC improved symptoms, pulmonary function tests, and HRCT findings in patients with PM/DM
	Niakan et al. (184)	No	4	Improved when treated with a combination of and cyclophosphamide after having become refractory to corticosteroid therapy alone
	Fries et al. (185)			CYC treatment alone is not an effective option in patients with PM
Etanercept	Muscle Study Group (121)	Yes	16	5 of 11 subjects with DM in the etanercept arm were successfully weaned off prednisone, with no major safety concerns
Infliximab	Labioche et al. (186)			A case report on a 63 years old with worsening PM refractory to conventional therapy treated with infliximab showed marked improvement in muscle strength and improvement in EMG studies and serum CK levels
	Hengstman et al. (187)	No	2	Both patients demonstrated a marked and sustained subjective and objective improvement without the occurrence of any side effects
	Hengstman et al. (188)	No		Infliximab (an antiTNF a agent) was a successful induction monotherapy in untreated PM/DM but was effective for a limited time only

*RCT, randomized control trial*.

Table 4 summarizes clinical presentation, laboratory investigations, therapies, and prognosis.

**Table 4:** **Idiopathic inflammatory myopathies (IIM), Summary**.

Disorder	Demographics	Clinical characteristics	Lab investigations	Biopsy findings	Associated conditions	Management/therapy	Prognosis
Dermatomyositis (DM)	Female > male > 40 years	Proximal muscle weakness. Erythematous rash, shawl sign, gottrons papules	CK normal or raised up to 50 times normal	MAC deposition in the microvasculature. B cells and CD4^+^ T cells in the perimyseal and perivascular areas	Interstitial lung disease, malignancy vasculitis	Corticosteroids, azathioprine, methotrexate MMF, IVIG, cyclophosphamide, rituximab	Good, 5-year survival rate approaches 70% with treatment
Polymyositis (PM)	Female > male > 40 years	Proximal muscle weakness insidious onset	CK levels increased up to 50 times normal	Cell mediated cytotoxicity mechanism CD8^+^ T cells, macrophages invade non-necrotic muscle fibers	Interstitial lung disease, malignancy, mixed connective tissue diseases, myocarditis	Corticosteroids, azathioprine, methotrexate MMF, IVIG, cyclophosphamide, rituximab	Good, with treatment. 5-year survival 70%
Sporadic Inclusion body myositis (sIBM)	Male > female > 40 years	Involvement of finer and wrist flexors and knee extensors. Proximal as well as distal muscle weakness, facial muscles may be involved	CK is normal or mildly increased up to 10 times normal	Inflammation mediated by macrophages and CD8^+^ T cells. Accumulation of beta amyloid and tau protein within fibers	Scleroderma, mixed connective tissue disease	No proven therapy. Trial of steroids and immunosupressives. Alemtuzumab is a promising drug.	Poor even with treatment. Increased functional disability
Necrotizing autoimmune myopathy (NAM)	Female > male	Proximal muscle weakness	CK mildly elevated up to 10 times normal. Anti SRP antibodies. Anti HMGCR autoantibodies	Macrophages invade necrotic fibers. MAC deposition in microvasculature	Malignancy, connective tissue diseases	Corticosteroids, azathioprine, methotrexate, MMF, IVIG, cyclophosphamide, rituximab	Good response to treatment

## Conclusion

Idiopathic inflammatory myopathies are a group of varied presentations and pathogenesis. Different classifications over time have enhanced the diagnostic yield. Enhanced pathological processing and imaging of the muscles have improved the diagnosis. Newer agents are being investigated for the therapy of IIM and may lead to better results. Early diagnosis and therapy remains the gold standard for optimal prognosis. sIBM has been grouped with IIM and may eventually be established a neurodegenerative disorder due to pathological features and poor response to immune modulating therapies. A standardized classification will help with the relatively consistent diagnosis among different neuromuscular specialist and be meaningful for randomized studies.

## Author Contributions

AM contributed to the body and design of the write up and made required changes. JK wrote the abstract, helped with initial edits, and constructed the tables. Prof. GH did the critical review, editing, and approved the final manuscript. Dr. MG provided the figures and the figure legends.

## Conflict of Interest Statement

The authors declare that the research was conducted in the absence of any commercial or financial relationships that could be construed as a potential conflict of interest. The reviewer RNB and handling Editor declared their shared affiliation, and the handling Editor states that the process nevertheless met the standards of a fair and objective review.

## Footnotes

^1^https://clinicaltrials.gov/ct2/show/NCT0234789

^2^https://clinicaltrials.gov/ct2/show/NCT01925209
